# Anxiety in Outdoor Experiential Education: Examining Predictors, Sources, and Implications for Program Design

**DOI:** 10.3390/bs15060777

**Published:** 2025-06-04

**Authors:** Curt Davidson, Hannah McConnell, Kelsie Sibbald, Brian Croft, Ryan Zwart

**Affiliations:** 1 Quinney College of Natural Resources, Utah State University, Moab, UT 84532, USA; 2 Indiana University Outdoor Adventures, Indiana University, Bloomington, IN 47405, USA; hmmcconn@iu.edu; 3 Haub School of Environment and Natural Resources, University of Wyoming, Laramie, WY 82071, USA; ksibbald@uwyo.edu; 4 Touch of Nature, University of Southern Illinois, Carbondale, IL 62901, USA; bcroft@siu.edu; 5 Department of Health and Human Performance, University of Tennessee, Chattanooga, TN 37403, USA; ryan-zwart@utc.edu

**Keywords:** anxiety, outdoor experiential education, stress, mental health, experiential learning

## Abstract

Pre-trip anxiety is a critical yet understudied factor influencing participation and engagement in Outdoor Experiential Education (OEE) programs. Anxiety can serve as both a motivator and a barrier, shaping participants’ willingness to engage in challenging activities. This study examines the sources, predictors, and temporal progression of pre-trip anxiety among OEE participants, with a focus on experience level, gender differences, and activity type. Using a cross-sectional design, data were collected from students and staff at two Midwestern universities across three time points leading to an OEE program. Measures included the State–Trait Anxiety Inventory (STAI) and a course-specific worry scale assessing concerns such as physical ability, social dynamics, and environmental risks. Results indicate that experience level can predict anxiety, with less experienced participants reporting higher levels of concern. Gender differences were also observed, with female participants exhibiting greater pre-trip anxiety, particularly in water-based activities. However, anxiety remained relatively stable across time intervals, suggesting that interventions may be effective at multiple stages before a program begins. These findings have practical implications for OEE design, including targeted pre-trip interventions, adjusted program marketing, and tailored support strategies to reduce barriers to participation and enhance student preparedness.

## 1. Introduction

Anxiety plays a dual role in Outdoor Experiential Education (OEE) programs, acting as both a motivator and a barrier to engagement. OEE relies on elements such as anxiety and risk, which are essential for fostering growth and resilience (Reed & Smith, 2023). However, rising anxiety levels among individuals have become a significant concern for OEE participants and practitioners (Barendse et al., 2023). Young adults, a key demographic, are particularly vulnerable, with increasing behavioral challenges and absenteeism reported (Davidson & Chau Rohn, 2023). This study investigates the root causes of pre-trip anxiety in OEE participants, examining how it manifests and evolves prior to program participation. The goal is to identify patterns that can enhance preparedness, reduce “no-shows”, and develop strategies for anxiety reduction before engaging in OEE experiences.

As such, this project was guided by four main research questions. First, we wanted to examine when, before an OEE experience, students might be feeling the most anxious about the upcoming experience. Second, we wanted to understand if participation in different activities might be more anxiety-inducing, e.g., whitewater paddling versus rock climbing. Third, we wanted to understand if prior experience with an activity would be an indicator of the amount of anxiety an individual was experiencing. Finally, we wanted to examine if staff who were facilitating the OEE experience had similar anxiety levels and triggers to the students.

OEE programs are designed to provide transformative experiences that foster personal growth, resilience, and a deepened connection with nature (Braun & Dierkes, 2017; D’Amato & Krasny, 2011; Ewert & Davidson, 2021). According to Reed and Smith (2023), these programs promote developmental and educational benefits but also pose unique challenges, including the onset of anxiety among participants. This project seeks to explore when, during the weeks leading up to an OEE course, anxiety manifests most significantly and the various sources of pre-trip anxiety that students might encounter before participating in such programs, highlighting concerns that could potentially affect their overall learning and positive outcome acquisition often associated with OEE programs.

### 1.1. Anxiety

Anxiety, a complex and multifaceted psychological state, has long been a subject of intense scholarly scrutiny. The term ’anxiety’ is often used to encompass a wide range of unpleasant emotions, including apprehension, dread, and unease (Salecl, 2004; Strongman, 1995; Zeidner & Matthews, 2010). Anxiety can be differentiated from fear, with the former being a future-focused, sustained emotional response to uncertainty, while the latter is a brief, present-focused reaction to a specific threat (Daniel-Watanabe & Fletcher, 2022). Importantly, not all anxiety is inherently negative or deleterious. Anxiety has long been identified as serving an adaptive function, preparing the individual to respond effectively to potential threats (Cattell & Scheier, 1958). This form of anxiety, known as eustress, can enhance performance and motivation, driving individuals to meet challenges and overcome obstacles (Aini, 2023; Smith & Merwin, 2021). In contrast, distress-inducing anxiety can have deleterious consequences, leading to a range of physiological and psychological symptoms, such as increased heart rate, elevated blood pressure, and feelings of apprehension (Hildrum et al., 2008; Millroth & Frey, 2021).

### 1.2. Theoretical Foundations

The foundations of anxiety, eustress, and distress are based on the work of Hans Selye, who differentiated between positive and negative stress (Selye, 2019). His concepts have significantly influenced our understanding of anxiety, performance, and well-being, informing this project’s framework. When designing experiential education programs, it is crucial to balance the stress participants experience with appropriate recovery time.

Several theories provide a framework for understanding the dual role of anxiety in Outdoor Experiential Education (OEE). This perspective highlights how anxiety can act as both a catalyst and a barrier to transformative learning. Anxiety often arises in OEE environments due to elements of novelty, uncertainty, and perceived risk (Davis-Berman & Berman, 2002). While these factors are crucial for transformative learning, they also require careful management of anxiety to enhance, rather than hinder, the learning process.

Kolb’s (2014) Experiential Learning Cycle highlights the role of anxiety in the learning process. When participants face new challenges during concrete experiences, anxiety often peaks, which can enhance their engagement in reflective observation and abstract conceptualization. However, if anxiety becomes too intense, it may disrupt the cycle, causing participants to withdraw or disengage before completing their learning (Braubach et al., 2017).

The balance between productive and counterproductive anxiety is highlighted by Csikszentmihalyi et al.’s (2014) concept of flow. Flow is an optimal state where anxiety signals that individuals are stretching their comfort zones, fostering meaningful growth. The adventure experience paradigm examines how perceived risk and competence create anxiety in Outdoor Experiential Education (OEE) activities (Harris et al., 2023; Priest & Martin, 1985). This dynamic influences whether participants convert anxiety into positive experiences or feel overwhelmed.

Together, these theoretical perspectives reveal that anxiety management is central to successful OEE outcomes. When participants can effectively navigate challenges through perceived competence, they can transform anxiety into focused attention and engagement, as Mitchell observed in the “mountain experience”, where climbers achieve flow through concentrated focus on immediate challenges (Melnikoff et al., 2022). This transformation occurs not from the danger itself but from the participants’ ability to exert mastery over perceived risks (Kirchner et al., 2008; Lu & Liao, 2012).

Understanding pre-course anxiety triggers is therefore critical to establishing conditions where anxiety serves its productive function in the learning process. Properly managed, anxiety becomes an integral component of the transformative potential of OEE, creating the necessary psychological conditions for participants to expand their capabilities and achieve meaningful personal growth. However, this requires careful program design that accounts for individual differences in anxiety responses and provides appropriate scaffolding to support participants in developing competence that matches perceived risks.

Beyond these situational triggers, individual differences significantly influence the level of anxiety experienced by participants in OEE programs. Several studies have explored how traits such as gender, age, and prior experience affect anxiety levels. For instance, earlier studies like those by Ewert (1989) and Onwuegbuzie (1995) suggest that women may experience higher levels of anxiety compared to men in these settings. However, other research, such as that by Baloglu (2001), indicates that there are no significant gender differences in anxiety levels within OEE contexts. Age is another factor, with younger participants typically experiencing more anxiety than their older counterparts, possibly due to their lesser experience in managing risk and uncertainty. Moreover, individuals with prior experience in OEE are found to have lower levels of anxiety, as familiarity with the activities often breeds confidence (Brey & Lehto, 2007).

The state of knowledge regarding anxiety in the context of outdoor education reveals a multifaceted understanding of its presence, management, and impact. Anxiety, often a central emotional response to perceived risks in outdoor activities, is both a challenge and a pedagogical tool in outdoor experiential learning. It is increasingly recognized that outdoor education settings can elicit both state anxiety, which is situational and temporary, and trait anxiety, a more stable and dispositional form of anxiety. These emotional states are triggered by physical, social, and psychological factors, such as fear of injury, social judgment, or self-doubt, often exacerbated by unfamiliar and challenging environments (Ewert, 1989; Reed & Smith, 2023).

Research has established that OEE programs often incorporate fear and anxiety intentionally, considering them essential for fostering resilience, problem-solving skills, and self-efficacy (Drebing et al., 1987). For instance, facilitators use fear as a developmental tool to push participants out of their comfort zones into a “stretch zone”, promoting learning through discomfort (Reed & Smith, 2023). However, this approach is not without criticism. Scholars have noted that not all participants respond positively to anxiety-inducing scenarios, as some may disengage, experience heightened fear responses, or feel marginalized when they cannot overcome these challenges (Reed & Smith, 2023).

### 1.3. Need for This Study

The exigency of this study emerges from a dual-faceted concern within OEE: the educational potential of anxiety juxtaposed against its capacity to hinder participation, stifle learning, and limit engagement. Anxiety’s rising prevalence, particularly following the COVID-19 pandemic, has become increasingly apparent amongst the college-aged demographic that OEE programs predominantly serve (Chang et al., 2021). This trend has been accompanied by escalating incidences of behavioral issues and absenteeism, posing significant challenges to practitioners (Davidson & Chau Rohn, 2023; Larson et al., 2023). Such developments necessitate a scholarly inquiry into the antecedents of anxiety within this context. This study aims to give OEE educators the insights needed to address these emotional barriers preemptively by investigating the factors contributing to pre-trip anxiety and their temporal dynamics. This, in turn, can enhance program retention, ensure student well-being, and uphold the transformative essence of OEE experiences. Thus, this research is poised to offer timely and actionable recommendations pertinent to contemporary OEE programming and the evolving needs of its participants.

## 2. Materials and Methods

### 2.1. Participants

Approval for this project was obtained from the host organization’s Institutional Review Board [IRB-2024-140]. This investigation employs a self-selection methodology wherein participants are drawn from individuals already enrolled in either a college-based freshman outdoor orientation program or a for-credit outdoor skill-based course at two major Midwestern universities. The selected outdoor programs involve a suite of activities that are intrinsically challenging and potentially anxiety-inducing, including backpacking, mountain biking, rock climbing, and canoeing. Further, each program utilized small-group membership and expert instruction from trained staff.

### 2.2. Instrument Development

The primary instrument for data collection is a contemporaneously adapted version of Quinn’s (1996) original instrument, refined to incorporate present-day stressors, such as digital disconnection and concerns over emergent stressors like active shooter situations. A full version of the instrument can be seen in Supplementary Materials. This modernized instrument was subjected to a pilot study within a graduate research methods course to ensure its reliability and appropriateness for the present investigation. In addition to the instrument asking about specific fears, the State–Trait Anxiety Inventory (STAI) was used to measure general levels of anxiety. This instrument is a validated measure of both state and trait anxiety and was used concomitantly to discern transient anxieties specific to the upcoming trip from the participants’ baseline anxiety propensities (Spielberger, 1989).

### 2.3. Data Collection Procedures

Survey dissemination occurred at three junctures: two weeks prior to the program commencement, one week prior, and on the initial day of the program. Subjects were each randomly assigned into one of the data collection time periods. They were prompted by the staff to complete the survey using a mobile-based platform during pre-trip classroom sessions or orientation meetings, ensuring a consistent administration protocol. Employing a cross-sectional design, this approach enabled the assessment of the trajectory of pre-trip anxiety, from its inception to its potential abatement or intensification, as the program start date drew closer. This modality was chosen for its standardization and efficiency in aggregating data for analysis.

- Current Anxiety (CA): A 20-item state anxiety scale was employed, which refers to a psychological framework distinguishing between state anxiety, a temporary and situation-specific emotional response to stress, and trait anxiety, a more stable, personality-driven tendency to experience anxiety across various situations. State anxiety fluctuates based on immediate circumstances (higher scores indicating higher levels of anxiety). Participants responded to items such as “I feel strained” or “I am tense” on a scale from 1 (Strongly Disagree) to 4 (Strongly Agree). The scale exhibited adequate levels of reliability (α = 0.92).
- Course Worry (CW): A 37-item course-specific worry scale was also administered. This scale used the following prompt: You have enrolled in an adventure course that will require you to be outdoors for multiple days. During this time, you will participate in various challenging activities with other students and staff. They were then asked to respond to specific items with themes of anxiety such as, “I am worried about my ability to perform” and “I am worried about being isolated”. Participants responded on a scale from 1 (Strongly Disagree) to 5 (Strongly Agree). The scale exhibited adequate levels of reliability (α = 0.95).

### 2.4. Data Analysis

As previously stated, we were interested in a few main research questions. First, we want to ascertain at which time interval students felt the most anxious (RQ1), whether activity type and experience level are related to anxiety scores (RQ2 and RQ3, respectively), and if there are differences between staff and student regarding the above effects (RQ4). Finally, we also wanted to determine what specific elements of an OEE experience was the source of the participants’ pre-course anxiety (RQ5). To assess data distribution, we conducted tests for normality, including the Shapiro–Wilk test, to determine whether the anxiety scores followed a normal distribution before applying parametric analyses. RQ1 was examined using a one-way ANOVA with the time interval group factor predicting course anxiety (CA) and course worry (CW) scores. ANOVA was used in this study to compare anxiety levels across multiple time points and activity types, allowing for the identification of significant differences while accounting for between-group variance in a statistically robust manner.

Regression analysis was used to examine the predictive relationships between anxiety levels and key variables such as experience level, gender, and course type, allowing for the identification of significant contributors to anxiety while controlling for other factors. For activity level, coherent groups were created to allow for sufficient sample sizes to run one-way ANOVA for the CA and CW outcomes. Finally, factorial ANOVAs and multiple regression with interaction terms included were used to test for the moderating effect of field course role (student vs. staff) on potential links between time interval/activity type/experience level and CA and CW scores, as well as the exploratory moderating effect of gender on these links as well. All analyses were conducted in SPSS Version 28.0. Interaction effects with continuous variables were conducted using the PROCESS macro (Hayes, 2017), which used 5000 bootstrapped confidence intervals to detect significant effects. Finally, mean scores for specific course worry elements are provided.

Finally, independent sample *t*-tests were used to compare anxiety levels between different course types (hard terrain vs. water-based activities) and participant roles (students vs. instructors) to determine whether significant differences existed between these groups. This approach allowed for a straightforward examination of mean differences in anxiety while accounting for variability within each group.

## 3. Results

Descriptive statistics can be found in Table 1. In order to preempt small cell sizes, course types were aggregated into two main groups: hard terrain (backpacking, rock climbing, mountain biking, outdoor leadership) and water activities (coastal kayaking, canoeing, whitewater kayaking/rafting). Participants were split relatively evenly across time points, and CA and CW scores were relatively low. The sample contained predominately students with a small subsample of instructors (*n* = 13). Basic correlations for certain variables of interest showed that experience level was significantly related to CA and CW outcomes—those with more experience tended to have lower levels of CA (r = −0.25, *p* = 0.004) and lower levels of CW (r = −0.21, *p* = 0.019). Instructors tended to have lower CW scores (r = −0.18, *p* = 0.038) and higher levels of experience (r = 0.51, *p* < 0.001). Expectedly, CA and CW scores were related to each other (r = 0.61, *p* < 0.001).

Next, the one-way ANOVAs determined if time interval was related to CA or CW scores. The results showed that there was no significant difference in scores for either the CA outcome [F (2, 117) = 0.77, *p* = 0.466] or the CW outcome [F (2, 117) = 1.12, *p* = 0.328]. This provided evidence as to RQ1, suggesting that CA and CW scores did not differ between time points. Independent sample *t*-tests showed that scores did not significantly differ between hard terrain and water activities for the CA outcome [t (128) = 0.05, *p* = 0.958; Cohen’s d = 0.01] nor for the CW outcome [t (128) = 0.71, *p* = 0.480; Cohen’s d = 0.14], giving clarity as to RQ2.

Multiple regression analysis showed that the effect for gender was significant (β = 0.18, *p* = 0.039) but was not significant for course type or role (|β|s < 0.05, *p*s > 0.649). Importantly, the effect of experience level on CA remained significant (β = −0.29, *p* = 0.004). For the CW outcome, a similar pattern emerged—however, although gender was once again a significant predictor (β = 0.24, *p* = 0.007), the effect of experience level was no longer statistically significant (β = −0.16, *p* = 0.104). These effects provide evidence relative to RQ3.

**Figure 1:**
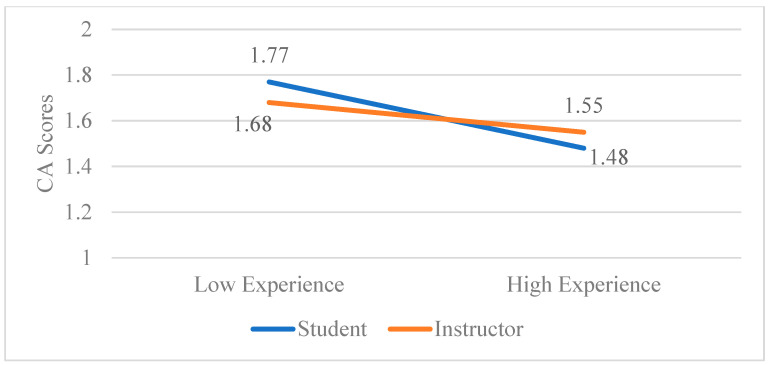
The role*experience interaction on course anxiety scores. *N* = 127.

As for RQ4, factorial ANOVA was used to determine if there were significant time*role or course type*role interactions for the CA and CW outcomes. For CA, the role*time term was not statistically significant (*p* = 0.267)—post hoc comparisons of simple effects confirmed no significant differences between roles at any of the three time points (all *p*s > 0.205), nor were there any significant differences between time frames for students (all *p*s > 0.328) or for instructors (all *p*s > 0.568). For CW, the role*time interaction was also not significant (*p* = 0.816). All follow-up comparisons were not significant, although the comparison for CW scores between students and instructors at time 2 was approaching significance (*p* = 0.104). An identical pattern of non-significance was found for the role*course type interactions and post hoc comparisons (all *p*s > 0.347). As for experience*role, although the interaction term was not significant (*p* = 0.671), simple slope effects showed a significant effect for students (β = −0.26, *p* = 0.005) but not for instructors (β = −0.10, *p* = 0.746). The interaction term was not significant (*p* = 0.977) for CW and simple slopes were not significant for students (β = −0.13, *p* = 0.155) nor for instructors (β = −0.16, *p* = 0.716). Below, Figure 1 depicts course anxiety scores comparing students versus instructors.

**Figure 2:**
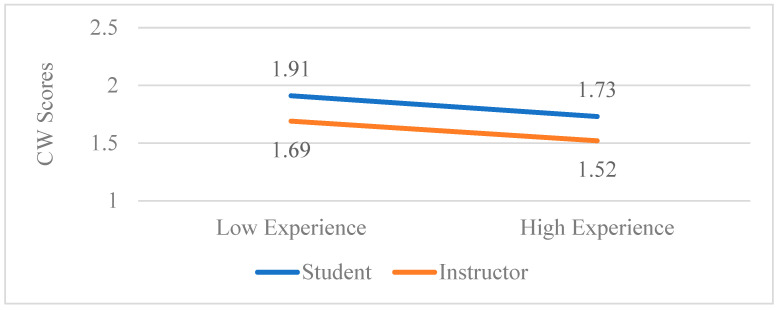
The role*experience interaction on course worry scores. *N* = 127.

Figure 2 (below) highlights the differences among staff and students on the aggregate score of “course worry”.

**Figure 3:**
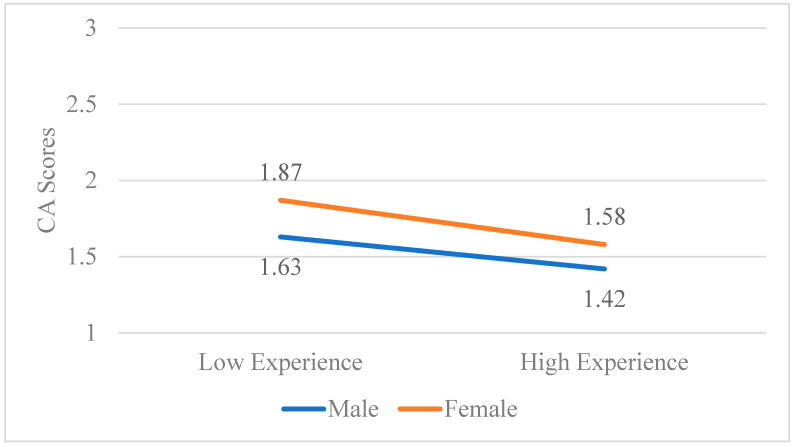
The gender*experience interaction on course anxiety scores. *N* = 127.

**Figure 4:**
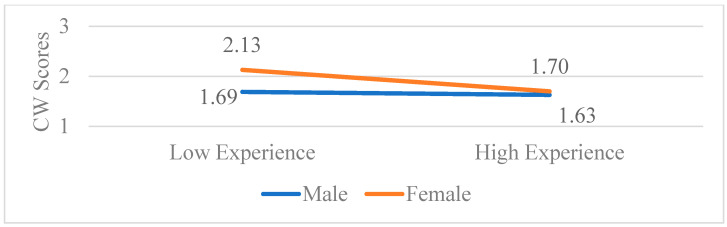
The gender*experience interaction on course worry scores. *N* = 127.

Exploratory analyses were conducted for gender on the aforementioned relationships. The gender*time interaction was not significant (*p* = 0.292), but simple effects showed that females had significantly higher CA scores at time 3 than males (*p* = 0.032). This pattern emerged for CW scores as well, with higher worry for females compared to males at time 3 (*p* = 0.023). No significant effects emerged for the gender*course type for CA (all *p*s > 0.138). For CW, females had higher CW scores in water sports compared with hard terrain courses (*p* = 0.047). Finally, the gender*experience interaction terms were not significant for CW or CA (*p*s > 0.104), but simple slopes showed a significant effect for females for CA (β = −0.30, *p* = 0.012) and for CW (β = −0.33, *p* = 0.004), while the effects for males were not significant (*p*s > 0.108). Effects for the gender*experience interaction on CA and CW can be found in Figure 3 and Figure 4, respectively. Mean scores for CA and CW scores across the three time points can be found in Table 2.

Finally, the analysis of mean anxiety scores revealed notable differences between students and staff across a range of pre-trip concerns. Among students, food-related worries emerged as the highest source of anxiety (M = 2.46), followed by concerns about social dynamics (M = 2.31) and personal hygiene (M = 2.32). These findings suggest that basic needs and interpersonal interactions are central to student anxieties prior to OEE participation. In contrast, staff reported lower overall levels of anxiety, with performance ability (M = 2.00) and environmental dangers (M = 2.08) ranking among their primary concerns. Both groups shared similar levels of anxiety regarding preparedness (students: M = 2.22; staff: M = 2.00) and physical ability (students: M = 2.15; staff: M = 2.00). Interestingly, perceived environmental dangers (students: M = 2.08; staff: M = 2.08) and concerns about injury (students: M = 2.11; staff: M = 2.00) were relatively low across both groups, suggesting confidence in the safety of the program structure. The full list of these anxieties can be viewed in Table 3. These patterns highlight the importance of addressing practical concerns, such as food and equipment preparation, alongside social and emotional support strategies, to alleviate student anxieties, while providing staff with training to bolster their confidence in facilitating activities.

## 4. Discussion

The findings from this study provide an important framework for understanding barriers to participation in OEE, complementing the work on motivations and anxiety for participation by Davidson and Chau Rohn (2023). Contrary to common assumptions, our results indicate that pre-trip anxiety remains relatively stable over time rather than escalating as the departure date approaches. This stability suggests that anxiety regarding OEE may be more trait-like in nature, or that participants reach a baseline level of concern that persists throughout the pre-trip period.

This finding aligns with theoretical perspectives from Salecl (2004) and Strongman (1995), who note that anxiety is fundamentally future-oriented, which is a finding still relevant in the literature (Li et al., 2024). The cross-sectional design of our study establishes a foundation for understanding how anxiety manifests in anticipation of OEE experiences and provides insights into optimal timing for interventions. Furthermore, the observed stability in anxiety levels suggests that existing pre-trip orientations and communications may not be effectively addressing participants’ concerns, indicating a need for more targeted engagement strategies.

While the temporal findings of this study were not statistically significant, they nevertheless contribute valuable insights to the corpus of knowledge on OEE. Notably, heightened anxiety can impair learning, supporting recommendations for educators to carefully consider the type and amount of information provided to learners before an OEE experience.

### 4.1. Practical Implications

Our findings challenge conventional approaches to addressing pre-trip anxiety by suggesting that interventions should not be concentrated immediately before departure. Instead, OEE practitioners should implement early and ongoing anxiety-reduction strategies throughout the pre-trip period. For example, addressing general anxieties approximately two weeks before a trip while focusing on immediate concerns, such as food availability, closer to the event may prove effective.

The research holds practical implications for how OEE programs are structured and marketed. An evidence-based understanding of anxiety timelines allows practitioners to strategically design interventions that address participants’ concerns from the outset. Program marketing and descriptions should be crafted to proactively address common sources of anxiety, such as concerns about food, social dynamics, or environmental dangers. Providing clear, detailed information about trip logistics and expectations can help students feel more comfortable attending pre-trip meetings and ultimately participating in the OEE course.

Our study reveals that experience levels significantly influence anxiety, with participants who have prior exposure to outdoor activities reporting lower levels of both general anxiety and course-specific concerns. This suggests that incorporating preparatory experiences, such as pre-program workshops or simulated activities, could help familiarize participants with potential challenges, thereby reducing anxiety and promoting confidence. OEE practitioners should consider designing experiences that align with participants’ comfort levels, such as prioritizing activities with lower perceived physical or social risks for less experienced groups. Content could center around the most anxiety-inducing components found in this study such as concerns around food, physical abilities, and social dynamics.

Since higher competence correlates with lower anxiety levels (Brey & Lehto, 2007; Ewert, 1989), programs should implement effective and efficient ways to boost confidence before or at the beginning of courses. Micro-activities, such as quick knot-tying exercises at the course onset, may boost self-efficacy, facilitate transition into the OEE experience, and lower anxiety levels. Confidence-inducing activities that occur before the trip may also lessen dropout rates.

Gender differences in pre-trip anxiety require further investigation, particularly regarding socialization patterns, stereotype threat, and prior outdoor exposure. Research suggests that women may experience higher anxiety in outdoor settings due to societal norms that promote risk aversion, whereas men are encouraged to take risks (Ewert, 1989; Baloglu, 2001). Additionally, stereotype threat and disparities in access to childhood outdoor recreational opportunities may contribute to these differences. While gender-informed interventions can be valuable, a more nuanced approach addressing these underlying factors may improve program design. By understanding these dynamics, OEE programs can create targeted strategies to address the root causes of gender disparities in anxiety.

The observed trends in anxiety among specific groups, including instructors versus students, suggest that facilitators and staff also benefit from targeted support and training to manage their own concerns. Addressing staff anxiety may indirectly enhance their ability to support participants effectively.

### 4.2. Future Research

Future research should explore how different types of anxiety—logistical, social, or performance-related—change over time, as this could improve interventions aimed at enhancing participant readiness and confidence. It is also important to assess how pre-trip anxiety develops leading up to the OEE experience, reflecting the impact of anticipatory stress. As previously stated, future research should also explore the gender-based differences highlighted briefly in these findings.

This study was also limited by access to various populations. Future studies should examine how age, program location, and activities all influence pre-course anxiety. As stated, this study focused primarily on college-aged participants. Anxiety may manifest in very different ways with young or adult OEE participants. Finally, future research should also include programs that do not offer any pre-trip meeting options to see how anxiety is different in those circumstances and programs.

Additionally, future studies should examine how anxiety influences overall participation and potentially contributes to the decline of OEE programs (Our Final Season. | Northwest Outward Bound, n.d.; REI Exits Experiences Business, n.d.). This line of inquiry is particularly relevant in an era where young people face unprecedented stressors, including global pandemics, civic unrest, and economic uncertainty. Understanding how experiential education can serve as a counterforce to these modern anxieties represents a valuable direction for future research.

### 4.3. Limitations

This study offers a valuable framework for understanding pre-trip anxiety in OEE programs; however, several limitations should be acknowledged. First, the context in which the study was conducted involved settings with pre-trip meetings that may have alleviated some participants’ concerns. This preparatory exposure could have influenced anxiety levels, potentially masking the full scope of pre-trip stress. Future research in environments that do not include such interventions would provide a clearer picture of how anxiety develops independently. Second, the sample composition primarily included college-aged students and a small number of instructors, limiting the generalizability of the findings. Broader demographic representation is necessary to determine whether anxiety trends hold true across different age groups and contexts. Third, although the study examined variables such as gender, prior experience, and activity type, it did not account for cultural or socioeconomic factors that may significantly shape perceptions of risk and anxiety. Including these dimensions in future studies would contribute to a more comprehensive understanding of pre-trip anxiety.

Finally, methodological limitations should be considered. The reliance on self-reported survey data introduces the potential for bias, and future studies would benefit from integrating observational data or physiological stress indicators to improve validity. Previous research on probability overestimation suggests that individuals with higher anxiety often overestimate the likelihood of negative events, which may contribute to the anticipatory stress observed in OEE participants (Bishop & Gagne, 2018; Gangemi et al., 2019). Gangemi et al. (2019) outline several cognitive restructuring techniques which have shown promise in reducing these overestimations and as a result improve anxiety regulation. By addressing these overestimations before the experience, through integrated cognitive-based interventions, participants may experience enhanced confidence and unnecessary barriers to engagement may be reduced. Additionally, the relatively small sample size constrained the power of the cross-sectional analysis; larger and more diverse samples are needed to strengthen the robustness and generalizability of the findings.

## 5. Conclusions

This study provides valuable insights into the dynamics of pre-trip anxiety in OEE programs, emphasizing its significance in shaping participant experiences and outcomes. The findings highlight the critical role of experience levels, gender differences, and activity types in influencing anxiety, offering actionable guidance for program design and facilitation. Specifically, the results suggest that preparatory experiences, targeted interventions, and tailored support strategies can effectively mitigate anxiety, creating an environment where participants feel confident and ready to engage.

While anxiety is an inherent part of OEE, often driving growth and learning, this study underscores the importance of balancing its facilitative and debilitating effects. By understanding the nuanced ways in which anxiety manifests, both as a stable pre-trip phenomenon and as a variable influenced by individual and contextual factors, OEE practitioners can implement and utilize this information to develop evidence-based strategies to support participants at every stage of their pre-trip experience. This will ultimately lead to more efficacious OEE courses and experiences for students. Future research should expand on these findings by exploring contexts without pre-trip meetings, examining anxiety across diverse populations, and integrating additional measures to capture a more comprehensive picture of its effects. By continuing to investigate and address pre-trip anxiety, the field of OEE can enhance its transformative potential, ensuring that participants are empowered to embrace challenges, overcome fears, and achieve meaningful personal and educational growth.

## Figures and Tables

**Table 1:** Descriptive statistics for the variables of interest.

	Current Anxiety	Course Worry	Experience Level
M (SD)	1.63 (0.49)	1.8 (0.60)	2.32 (1.27)
Time Interval	Time 1	Time 2	Time 3
	28	53	39
Role	Student	Instructor	
	118	13	
Course Type	Hard Terrain	Water Activity	
	94	36	

Note: *N* = 131.

**Table 2:** Course anxiety and course worry scores from the descriptive statistics across time points.

		CA Scores			CW Scores	
Group	Time 1	Time 2	Time 3	Time 1	Time 2	Time 3
Males	1.56 (0.48)	1.66 (0.59)	1.48 (0.35)	1.61 (0.44)	1.89 (0.54)	1.57 (0.48)
Females	1.55 (0.34)	1.74 (0.54)	1.82 (0.61)	1.89 (0.54)	1.93 (0.61)	2.02 (0.78)
Students	1.54 (0.036)	1.74 (0.55)	1.63 (0.50)	1.80 (0.57)	1.97 (0.58)	1.77 (0.65)
Instructors	1.75 (0.0.73)	1.48 (0.38)	1.00	1.33 (0.29)	1.60 (0.41)	1.00
Hard Terrain	1.56 (0.04)	1.68 (0.51)	1.68 (0.52)	1.75 (0.56)	1.91 (0.57)	1.83 (0.67)
Water Terrain	N/A	1.79 (0.66)	1.56 (0.49)	N/A	1.91 (0.64)	1.69 (0.65)

**Table 3:** Mean scores from the descriptive statistics of specific anxiety triggers before an OEE course.

Student Anxiety	M	Staff Anxiety	M
Food	2.46	Social Dynamics	2.31
Hygiene	2.32	Environmental Dangers	2.08
Ability to Perform	2.24	Performance Ability	2.00
Preparedness	2.22		
Physical Ability	2.15		
Injury	2.11		
Being Uncomfortable	2.09		
Environmental Dangers	2.08		
Lack of Free Time	2.06		
Equipment	2.03		

Note: We used an arbitrary cutoff mean score of 2.00 for reporting purposes.

## Data Availability

Data available upon request. Please contact the corresponding author for data access.

## Supplementary Materials

The following supporting information can be downloaded at: https://www.mdpi.com/article/10.3390/bs15060777/s1, Anxiety Questions.
