# Supplementary material for: Anxiety in Outdoor Experiential Education: Examining Predictors, Sources, and Implications for Program Design
Source: Behav Sci (Basel). 2025 Jun 4;15(6):777. doi: 10.3390/bs15060777 (PMC12189140; doi:10.3390/bs15060777)
Supplement: Supplementary file 1 [file behavsci-15-00777-s001.zip › behavsci-3524799-supplementary.pdf]

Anxiety Questions

Please indicate your role in the field course. Instructor/Student
In which course are you participating?
What is your level of experience with the activity you will be participating in during the outdoor program?
If you are a student, what is your class standing?
How old are you?
How do you describe yourself?
Choose one or more races that you consider yourself to be
Fill in the response to the right of the statement to indicate how you feel at this moment. (1-5)

- I feel calm
- I feel secure
- I am tense
- I feel strained
- I feel at ease
- I feel upset
- I am presently worrying over possible misfortunes
- I feel satisfied
- I feel frightened
- I feel comfortable
- I feel self confident
- I feel nervous
- I am jittery
- I feel indecisive
- I am relaxed
- I feel content
- I am worried
- I feel confused
- I feel steady
- I feel pleasant
- I am worried about feeling out of control
- I am worried about being unsafe
- I am worried about the social dynamics
- I am worried about the environmental dangers
- I am worried about my ability to perform
- I am worried about the course not meeting my expectations
- I am worried about being isolated
- I am worried about the food while on my trip
- I am worried about the equipment I will have available
- I am worried about the staff
- I am worried about becoming injured
- I am worried about being uncomfortable
- I am worried about my hygiene
- I am worried about the living conditions
- I am worried about being harassed by others on the trip
- I am worried about my emotional well-being

I'm worried about my physical strength and endurance
I'm worried about how cognitively challenging this course will be
I am worried about how prepared I am
I worry about becoming sick
I am worried about being without my electronic devices
I am worried about my personal information being shared
I am worried about being without cellphone service
I am worried about being cyber-bullied after online content is released about the trip
I am worried about the ecological footprint of the course such as the impact of activities on the environment
I am worried about cultural appropriation or insensitivity in themes or activities
I am worried about the spread of infectious diseases, such as COVID-19, and the adequacy of health measures
I am worried about being included or accommodated due to gender identity, sexual orientation, or disability
I am worried about a polarized social and political environment that could manifest in conflicts during group activities
I am worried about an active shooter event
I am worried about terrorism while at the outdoor program
I am worried about over-scheduling and a lack of free time
I am worried about being constantly monitored or watched, even in seemingly private spaces like cabins or tents
I am worried about the physical accessibility of outdoor environments for those with mobility issues
I am worried about the cost of the camp or activity
I thought about not going on the outdoor trip
I am worried about boredom while on the trip

Is there anything not addressed in the survey that is making you feel anxious going into this field course?
